# The First Turkish Case of Hypoparathyroidism, Deafness and Renal Dysplasia (HDR) Syndrome

**DOI:** 10.4274/jcrpe.1874

**Published:** 2015-06-03

**Authors:** Hakan Döneray, Takeshi Usui, Avni Kaya, Ayşe Sena Dönmez

**Affiliations:** 1 Atatürk University Faculty of Medicine, Department of Pediatric Endocrinology, Erzurum, Turkey; 2 Kyoto Medical Center, National Hospital Organization, Clinical Research Institute, Kyoto, Japan; 3 Atatürk University Faculty of Medicine, Department of Pediatrics, Erzurum, Turkey

**Keywords:** HDR syndrome, infant, p.R367X mutation

## Abstract

Hypoparathyroidism, deafness and renal dysplasia (HDR) syndrome is an autosomal dominant genetic disorder characterized by hypoparathyroidism, sensorineural deafness and renal dysplasia. We herein present the first Turkish patient with HDR syndrome, who has a p.R367X mutation. This report indicates that p.R367X is not a mutation specific for the Far Eastern populations and also that urological findings in infants with hypoparathyroidism should be carefully examined because clinical findings relating to the p.R367X mutation may show a variable age of onset.

## INTRODUCTION

Hypoparathyroidism, deafness and renal dysplasia (HDR) syndrome is an autosomal dominant genetic disorder characterized by hypoparathyroidism, sensorineural deafness and renal dysplasia (1). Mutations in GATA3 gene located on the short arm of chromosome 10 are responsible for this syndrome. p.R367X is a mutation that causes a prematurely terminated protein with loss of basic amino acids in the flanking ZnF2 domain of GATA3 protein. To date, four HDR cases with heterozygous p.R367X mutation have been reported. Three of these cases were Japanese and Chinese (2,3,4). We herein present the first Turkish patient genetically diagnosed with HDR syndrome, who has the same mutation. This report suggests that the p.R367X is not a mutation specific for the Far East populations and demonstrates that hypoparathyroidism and sensorineural deafness may display clinical variability, while the renal manifestation is limited to proteinuria and haematuria.

## CASE REPORT

A 2-month-old Turkish male infant, the second child of a non-consanguineous marriage, was referred to our pediatric clinic with a history of an afebrile seizure. The patient was born at 40 gestational weeks by vaginal delivery after an unremarkable pregnancy. There was no family history of deafness or renal insufficiency. On admission, his vital signs were within normal limits. Weight, length and head circumference were 4470 g (25th percentile), 59 cm (50th percentile) and 39 cm (50th percentile), respectively. Physical examination findings were unremarkable. The low serum calcium (Ca) (6.4 mg/dL; normal range, 8.8-10.3 mg/dL), high serum phosphate (7.2 mg/dL; normal range, 3.8-6.5 mg/dL) and inappropriately low serum parathyroid hormone (PTH) (7 pg/mL; normal range, 20-65 pg/mL) levels of the patient led to a diagnosis of hypoparathyroidism. Serum alkaline phosphatase (ALP) and 25-hydroxy vitamin D [25(OH)D] levels were normal (388 U/L; normal range, 145-420 U/L and 22 ng/mL; normal range, 20-75 ng/mL, respectively). Urinalysis showed trace proteinuria and haematuria. Serum creatinine (Cr) level was high with 1.3 mg/dL (normal range, 0.2-0.4 mg/dL), while blood urea nitrogen was normal. Serum Cr level, proteinuria and haematuria persisted as 0.6-1.2 mg/dL, trace/(1+) and trace/(1+), respectively. Whole blood count, serum electrolytes including sodium, potassium and magnesium, liver tests and spot urine Ca/Cr ratio were unremarkable. The thymus gland was present on the neck ultrasonography. Urinary ultrasonography, diethylenetriamine-pentaacetic acid, echocardiographyand auditory function tests were normal. Based on presence of hypoparathyroidism and renal findings, a diagnosis of HDR syndrome was made. The tetanic seizures were controlled by intravenous Ca gluconate, followed by treatment with elementary Ca (50 mg/kg/day) and calcitriol (0.25 μ/d). The parents’ serum Ca, P, ALP, PTH and 25(OH)D levels were 9.2 and 9.4 mg/dL, 3.7 and 3.8 mg/dL, 323 and 332 U/L, 25 and 35 pg/mL and 28 and 32 ng/mL in the mother and the father, respectively.

### Molecular Genetic Analysis

**Figure 1:**
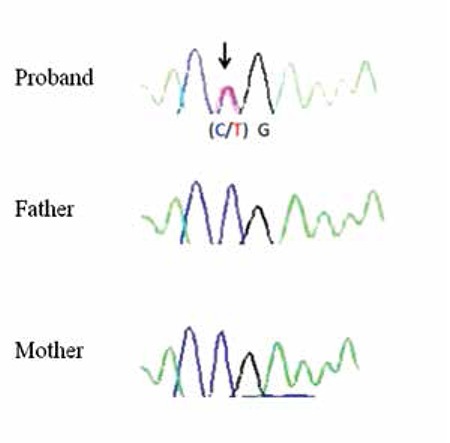
Electropherogram showing the heterozygous p.R367X mutation identified in the proband. The mutation was not detected in the mother’s and father’s samples. A black arrow points to the c.1099C>T mutation which results in substitution of a termination signal instead of arginine at residue 367 (p.R367X).

Sequence analysis of coding and flanking intronic regions of the GATA3 gene was performed. The results showed a nonsense mutation (p. R367X; c.1099C>T). The C>T mutation at nucleotide 1099 in exon 6 of the GATA3 resulted in substitution of a termination signal instead of arginine at codon 367. The patient’s parents showed wild type sequences (Figure 1).

## DISCUSSION

The human GATA3 gene is located on chromosome 10p14 and consists of six exons (5). This gene encodes a 444-amino-acid transcription factor that contains two zinc finger [N-terminal (ZnF1) and C-terminal (ZnF2)] and two transactivating domains (6). ZnF2 domain is essential for DNA-binding, whereas ZnF1 domain stabilizes this binding and physically interacts with other multitype zinc finger proteins such as the Friends of GATA (FOG) (3). GATA3 mutations can be divided into three groups. The first group includes the mutations affecting the ZnF2 domain. They result in a loss of DNA-binding and represent about 90% of detected mutations. The second group is defined by a decrease of DNA-binding affinity due to the lack of ZnF1 domain function. The third group have mutations characterized by normal DNA-binding and affinity but conformational changes of GATA3 protein or loss of interaction with FOG (7). The p.R367X mutation that we determined in our patient belongs to the first group and causes a prematurely terminated protein with loss of basic amino acids in the flanking ZnF2 domain. Four HDR cases with heterozygous p.R367X mutation have been reported to date (Table 1). Like our patient, no mutations were found in the reported patients’ parents. This situation suggests that the mutation is de novo or results from a germinal mosaicism in either the father or the mother (4). Patients with p.R367 mutation have clinical variability in terms of age of onset as well as intensity and diversity of findings.

As observed in Table 1, cases 1, 3 and 4 were Japanese and Chinese. To the best of our knowledge, our patient is the first Turkish case genetically diagnosed to have HDR syndrome. This finding suggests that the p.R367X is not a mutation specific for the Far Eastern populations.

GATA3 gene is expressed in the central and peripheral nervous system, inner ear, thymus, parathyroid, liver, kidney and hematopoietic cell lineage (8). Parathyroid glands, inner ear and kidney are the most frequently affected organs due to GATA3 gene mutations. Hypoparathyroidism in patients with HDR syndrome displays the widest individual variability, ranging from asymptomatic hypocalcemia to paresthesias, muscular aching and a frank tetanic picture, with low, normal or even slightly elevated serum PTH levels (9). The age of onset is widely variable. Our case is the youngest patient who developed hypoparathyroidism among the reported cases with p.R367X mutation (Table 1).

The clinical feature of early-onset sensorineural deafness is the most completely penetrant aspect of the HDR syndrome. Diagnosis can be facilitated and anticipated by the observation of the variant hearing problem in a patient with hypoparathyroidism. It is unknown to what extent the deafness is caused by defects in the peripheral and/or central auditory systems. However, both types of defect may contribute to hearing loss because GATA3 gene is prominently expressed during development of the ear as well that of the brain (10). The sensorineural deafness is usually bilateral, although the hearing loss may vary in its severity (1). The youngest case in terms of developing sensorineural deafness among the reported patients with p.R367X mutation was younger than 1 year. The onset in the other cases was during childhood. Cases 2 and 3 had bilateral sensorineural deafness and cases 1 and 3 were using hearing aids. Our patient showed no sensorineural deafness as yet. These findings suggest that the sensorineural deafness in patients with p.R367X mutation is variable in age of onset.

The renal phenotype of HDR syndrome is more variable. Renal manifestations may be uni-or bilateral and can include renal cysts, renal agenesis, renal dysplasia and vesico-ureteric reflux, variably represented even in affected members of the same family (1,2). Patients with p.R367X mutation have no major renal abnormalities (Table 1). Cases 1 and 3 had proteinuria and haematuria, suggestive of renal dysplasia. Our patient is the youngest patient who developed renal manifestations among the reported cases. In addition to proteinuria and haematuria, he also had a high serum Cr level which persisted between 0.6 and 1.2 mg/dL. The findings suggest that the p.R367X mutation does not cause major renal manifestations but lead to a high serum Cr level as well as to haematuria and proteinuria even in the infancy period.

In conclusion, we report the first Turkish case of a patient with HDR syndrome, who is also the youngest among the reported patients with p.R367X mutation. This report indicates that the p.R367X mutation is not specific for the Far Eastern populations. p.R367X mutation demonstrates a variable age of onset of hypoparathyroidism, sensorineural deafness and renal abnormalities. This mutation is associated with proteinuria and haematuria but not major renal abnormalities. However, urological findings in infants with hypoparathyroidism should be carefully examined.

## Figures and Tables

**Table 1:**
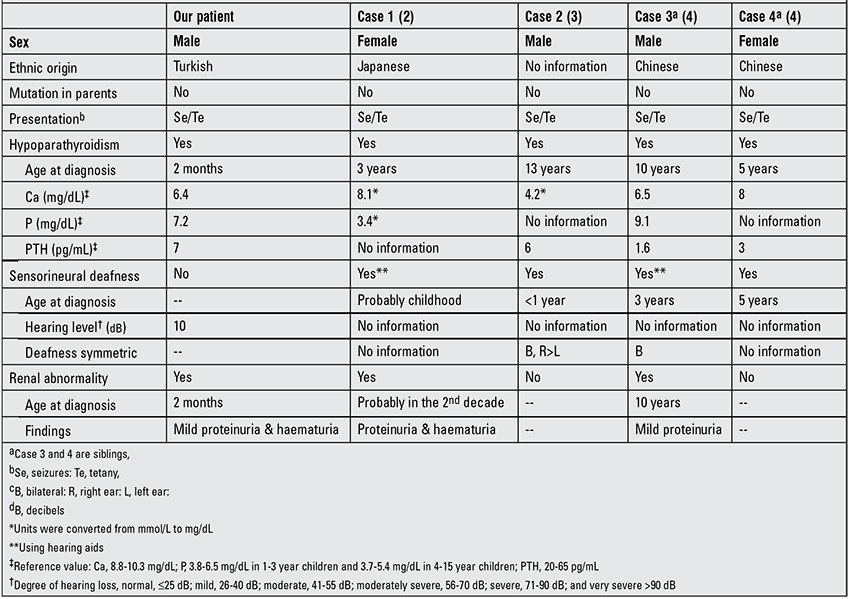
Clinical and laboratory characteristics of our patient and other reported cases with p.R367X mutation.
